# The SIFIPAC/WSES/SICG/SIMEU guidelines for diagnosis and treatment of acute appendicitis in the elderly (2019 edition)

**DOI:** 10.1186/s13017-020-00298-0

**Published:** 2020-03-10

**Authors:** Paola Fugazzola, Marco Ceresoli, Vanni Agnoletti, Ferdinando Agresta, Bruno Amato, Paolo Carcoforo, Fausto Catena, Osvaldo Chiara, Massimo Chiarugi, Lorenzo Cobianchi, Federico Coccolini, Alessandro De Troia, Salomone Di Saverio, Andrea Fabbri, Carlo Feo, Francesco Gabrielli, Angela Gurrado, Angelo Guttadauro, Leonardo Leone, Daniele Marrelli, Luca Petruzzelli, Nazario Portolani, Francesco Paolo Prete, Alessandro Puzziello, Massimo Sartelli, Giorgio Soliani, Mario Testini, Salvatore Tolone, Matteo Tomasoni, Gregorio Tugnoli, Pierluigi Viale, Monica Zese, Offir Ben Ishay, Yoram Kluger, Andrew Kirkpatrick, Luca Ansaloni

**Affiliations:** 1 General and Emergency Surgery Department, Bufalini Hospital, Viale Ghirotti 286, 47521 Cesena, Italy; 2 General Surgery Department, Milano-Bicocca University, School of Medicine and Surgery, Monza, Italy; 3 Intensive Care Unit, Bufalini Hospital, Cesena, Italy; 4 General Surgery, Civil Hospital - ULSS19, Adria, Italy; 5 Department of Clinical Medicine and Surgery, University of Naples “Federico II”, Naples, Italy; 6 Department of Surgery, S. Anna University Hospital and University of Ferrara, Ferrara, Italy; 7 Emergency and Trauma Surgery, Maggiore Hospital, Parma, Italy; 8 Emergency and Trauma Surgery, Niguarda Hospital, Milan, Italy; 9 Emergency Surgery Unit, State University of Pisa, Cisanello Hospital, Pisa, Italy; 10 Department of General Surgery, Fondazione IRCCS Policlinico San Matteo, Pavia, Italy; 11 Colorectal Unit, Addenbrooke’s Hospital, Cambridge University Hospitals NHS Foundation Trust, Cambridge, UK; 12 Department of Emergency Medicine, Morgagni-Pierantoni Hospital, Forlì, Italy; 13 Department of Biochemical Sciences and Human Oncology, University of Medical School “A. Moro” of Bari, Bari, Italy; 14 General and Oncological Surgery, Filippo Neri Hospital, Rome, Italy; 15 Department of Medicine, Surgery and Neurosciences, University of Siena, Siena, Italy; 16 Department of Emergency Surgery, Città della Salute e della Scienza University Hospital, Torino, Italy; 17 Department of Clinical and Experimental Sciences, Surgical Clinic, University of Brescia, Brescia, Italy; 18 Endocrine, Digestive and Emergency Surgery Department, University of Medical School of Bari, Bari, Italy; 19 General Surgery, Salerno University, Salerno, Italy; 20 Department of Surgery, Macerata Hospital, Macerata, Italy; 21 General, Mininvasive and Bariatric Surgery Unit, Department of Advanced Medical and Surgical Sciences, University of Campania Luigi Vanvitelli, Caserta, Italy; 22 Trauma Surgery Unit, Maggiore Hospital Regional Emergency Surgery and Trauma Center, Bologna Local Health District, Bologna, Italy; 23 Operative Unit of Infectious Diseases, S. Orsola-Malpighi University Hospital, Bologna, Italy; 24 Division of General Surgery, Rambam Health Care Campus, Haifa, Israel; 25 Departments of General Acute Care, Abdominal Wall Reconstruction and Trauma Surgery, Foothills Medical Centre, Calgary, Alberta Canada

**Keywords:** Appendicitis, Elderly, Appendectomy, Surgery in elderly

## Abstract

The epidemiology and the outcomes of acute appendicitis in elderly patients are very different from the younger population. Elderly patients with acute appendicitis showed higher mortality, higher perforation rate, lower diagnostic accuracy, longer delay from symptoms onset and admission, higher postoperative complication rate and higher risk of colonic and appendiceal cancer. The aim of the present work was to investigate age-related factors that could influence a different approach, compared to the 2016 WSES Jerusalem guidelines on general population, in terms of diagnosis and management of elderly patient with acute appendicitis. During the XXIX National Congress of the Italian Society of Surgical Pathophysiology (SIFIPAC) held in Cesena (Italy) in May 2019, in collaboration with the Italian Society of Geriatric Surgery (SICG), the World Society of Emergency Surgery (WSES) and the Italian Society of Emergency Medicine (SIMEU), a panel of experts participated to a Consensus Conference where eight panelists presented a number of statements, which were developed for each of the four topics about diagnosis and management of acute appendicitis in elderly patients, formulated according to the GRADE system. The statements were then voted, eventually modified and finally approved by the participants to the Consensus Conference. The current paper is reporting the definitive guidelines statements on each of the following topics: diagnosis, non-operative management, operative management and antibiotic therapy.

## Background

After adolescence, the incidence of acute appendicitis (AA) decreases with increasing of age [1]. Among patients presenting with acute abdominal pain in the Emergency Department (ED) about the 15% of patients older than 50 years old will have a final diagnosis of acute appendicitis, compared to nearly 30% of younger patients [2]. However, the epidemiology and the outcomes of acute appendicitis in elderly patients are very different from the younger population. First of all, in the face of a decrease in incidence, appendicitis in elderly patients is burdened by a significantly higher mortality [3] which reaches 8% among patients older than 65 years [2], compared to a rate ranging between 0 and 1% among younger patients. In a large observational study on 164.579 patients with acute appendicitis, an age older than 65 was a significant risk factor for mortality at multivariate analysis [3].

Furthermore, according to almost all authors, elderly patients were significantly more likely than other age groups to have complicated appendicitis with perforation or abscess. The complicated appendicitis rate ranges from 18 to 70% [2, 4–21] (compared to a rate ranging from 3 to 29% among patients younger than 65 years old). The reason for this high risk of perforation could be the vascular sclerosis that the vermiform appendix develops in elderly patients and the narrowing of the lumen by fibrosis. In these patients, the muscular layers are infiltrated with fat and there is a structural weakness with tendency towards early perforation [6].These finding, together with the delay of the diagnosis and of the treatment, could explain a more aggressive course of the disease in this population.

Another finding among elderly population with acute appendicitis is the lower rate of correct pre-operative diagnosis compared to younger population [4, 8, 9, 22],with a reported diagnostic accuracy (defined as the percentage of removed appendices with a histologic diagnosis of acute appendicitis from the total number of performed appendectomies) of 64% in patients over 65 years compared to 78% in other age groups (p > 0.01) [9].

Furthermore, in almost all the included studies, the average time from symptoms onset to admission and from admission to theatre was greater in older patients than in younger ones [2, 6, 8, 12, 23, 24].

Focusing on appendectomy, compared to young patients, elderly patients are burdened by a higher post-operative mortality [21, 25], a higher post-operative morbidity [12, 21], a longer length of stay [12], a longer operative time [12], a lower laparoscopic appendectomy rate [12, 14, 20] and a higher risk to receive more complex procedures [14]. In a large Swedish study [25] on more than 117,000 patients, the case fatality rate after appendectomy was strongly influenced by age with a threefold increase for each decade of age, reaching more than 16% in the nonagenarians.

Finally, the complication rate in elderly patients with negative appendectomy was significantly higher than in younger patients (25% vs 3%, *p* < 0.05) [2].

Despite acute appendicitis being more common in children and young adults, with the ageing of the western population, in the next years acute appendicitis in elderly patients will probably become more common. Since the lack of dedicated guidelines for elderly patients with acute appendicitis, the Italian Society of Surgical Pathophysiology (SIFIPAC) along with the Italian Society of Geriatric Surgery (SICG), the World Society of Emergency Surgery (WSES) and the Italian Society of Emergency Medicine (SIMEU) decided to develop the first evidence-based clinical guidelines for the management of acute appendicitis in elderly patients.

## Material and methods

In January 2019, the Italian society of Surgical Pathophysiology (SIFIPAC) along with the Italian Society of Geriatric Surgery (SICG) and the World Society of Emergency Surgery (WSES) nominated a scientific committee for the development of the guidelines for the diagnosis and the treatment of acute appendicitis in elderly patients.

Several definitions of elderly patients exist in literature with no clear and definite criteria; generally, most of the researches consider as elderly, all the patients with more than 65 years, but significantly heterogeneity exists. Moreover, the WHO has recently published new age cut-off for elderly, 75 years. The definition of elderly could not be based only on the chronological age but should be based on several factors determining the biological age. These factors are difficultly measured and no clear and objective definitions are available. For these reasons, we decided to define “elderly” as patients with more than 65 years.

The scientific committee defined four areas of interest: diagnosis, non-operative management, operative management and antibiotic therapy; for each interest areas were defined several questions, developed according to the PICO model. A systematic review of the available literature was made through an electronic bibliography search on PubMed and EMBASE. Two independent researchers were assigned to each area of interest. Each group during the study period analysed the available literature and according to the GRADE methodology developed the answer to the questions grading the quality of evidences and assigning the strength of the recommendation [26]. The quality of evidence was assessed and classified, according to the GRADE, in four levels: high, moderate, low and very low; the consequent recommendations were made based on the level of evidence and were classified in two levels: strong recommendation in favour or against and weak recommendation (suggestion) in favour or against.

Each proposed statement, along with the results of the systematic review of the literature, was illustrated and discussed during the plenary assembly of the XXIX SIFIPAC National Congress, held in Cesena, Italy, on May 3rd, 2019, with the participations of members of the SIFIPAC, the SIGC, the SIMEU and the WSES. Each statement was then voted by the audience and was approved if it reached at least 80% of positive votes; in case of discordance, the statement was improved and modified in order to reach the approval by the assembly.

## Results

### Diagnosis

1. Have existing clinical scoring systems sufficient diagnostic accuracy for the diagnosis of acute appendicitis in elderly patients?
  The Alvarado score is the most extensively studied score. Its validity on adult and children patients has been summarised in a recent meta-analysis [27] including 5960 patients in 29 studies. According to Ohle et al., the performance of the score is dependent on the cut-off value: a clinical cut-off score of less than five can be applied to “rule out” appendicitis with a sensitivity of 99% (95% CI 97–99%) and a specificity of 43% (36–51%).
  According to the Jerusalem guidelines [28] in adult patients, the Alvarado score (with cut-off score < 5) is sufficiently sensitive to exclude acute appendicitis, but it is not sufficiently specific in diagnosing acute appendicitis.
  However, Alvarado score was developed based on the presentation pattern, clinical and laboratory variables of a young population (mean age 23.4 − 25.9) [29].

A recent prospective interventional study and nested randomised trial [30] stated that the appendicitis inflammatory response (AIR score)-based risk classification can safely reduce the use of diagnostic imaging and hospital admissions in patients with suspicion of appendicitis, but this study is based on general population.

Few studies evaluated the applicability of existing appendicitis scores in elderly population [5, 31]. One retrospective study [5] on 96 patients with more than 65 years showed that the use of the Alvarado scoring system, with a cut-off of 5, maintains reliability in elderly patients. In fact, the vast majority of patients with pathologically confirmed appendicitis (86.6%) had an Alvarado score ranging from 5 to 8, with the 40% scoring either 5 or 6. According to these data, Alvarado scores ranging from 5 to 10 should correspond to high risk of appendicitis in the elderly. Another retrospective study [31] on 41 patients older than 65 years old, showed an area under the curve (AUC) of the Alvarado score for these population of 96.9% with 100% negative and positive predictive values of the two cut-off points of 3 and 6.At the light of the absence of high quality evidences dedicated to the elderly, after the discussion, the panel of experts could not make a strong recommendation; Alvarado score is suggested for excluding appendicitis, but not for diagnosing it, in elderly patients, with a conditional recommendation based on low quality evidences.

*Statement 1.1*. We suggest the use of scoring systems for excluding acute appendicitis in elderly patients with a low-probability score (*Conditional recommendation, low quality evidence*).

*Statement 1.2*. We suggest against basing the diagnosis of acute appendicitis in elderly patients only on scoring systems (*Conditional recommendation, low quality evidence*).

2. Could the diagnosis of acute appendicitis be based only on clinical signs and symptoms in elderly patients?
  In adult patients, laboratory tests of the inflammatory response and the clinical descriptors of peritoneal irritation and migration of pain are the strongest discriminators and should be included in the diagnostic assessment of patients with suspected appendicitis [28].

According to most included studies, among elderly patients, there is a lower rate of correct pre-operative diagnosis of acute appendicitis compared to younger population [4, 8, 9, 22].

Furthermore, in almost all the included studies, the average time from symptoms onset to admission and from admission to theatre was greater in older patients than in younger ones [2, 6, 8, 12, 23, 24].

There is still controversy on whether the presentation of appendicitis in the elderly patients differs significantly from those in the younger age groups [5–7].

According to some authors [5, 24] the typical triad of migrating right lower quadrant pain of short duration, fever and leucocytosis is infrequently observed. Many elderly patients with acute appendicitis have signs and symptoms consistent with ileus or bowel obstruction [2, 8]. Tenderness in the right lower quadrant, nausea and vomiting are common [8]. The reported rate of the presence of fever ranges from 30 to 80% [2, 5, 8]. However, only a minority of the patients has all of typical signs and symptoms together [8].

According to other authors, appendicitis does not present atypically in older patients. On the contrary, symptoms and signs reflect the severity of the abdominal disease, the delay of hospital admission and the high rate of perforations [2]. Indeed, significantly more signs and symptoms of peritonitis (abdominal distension, generalised tenderness and guarding, rebound tenderness, palpable abdominal mass) are recorded among older patients [2]. Probably, comorbidity and concurrent medication may further complicate the diagnosis.

*Statement 2*. In elderly population, we recommend against basing the diagnosis of acute appendicitis only on patient's clinical signs and symptoms (*Strong recommendation, low quality evidence*).

3. Have laboratory tests sufficient diagnostic accuracy for the diagnosis of acute appendicitis in elderly patients?

According to Andersson [32], in general population, appendicitis is likely when two or more inflammatory variables are increased and unlikely when all are normal. Furthermore, Yu et al. [33] found that procalcitonin has great diagnostic value in identifying complicated appendicitis (AUC value 0.94).

However, according to some studies, laboratory tests have no sufficient diagnostic accuracy for the diagnosis of acute appendicitis in elderly patients [34]. On the contrary, other studies showed as leukocyte response is not affected by age, and a significantly greater proportion of older patients had a raised white cell count compared with younger patients [2].

In a series of 83 consecutive elderly patients operated on for a clinical suspicion of acute appendicitis, although elevated leukocyte count and CRP value cannot effectively establish the diagnosis of acute appendicitis, unelevated values excluded it with a 100% negative predictive value [35].

According to some studies [13], a high CRP value in elderly patients with acute appendicitis, could be a suspect index for the existence of a perforation (AUC 0.811 with the cut-off of 101.9 mg/l). Shin et al. showed, as the delta neutrophil index, that measures of the fraction of immature granulocytes in the circulation is the only independent marker that can significantly predict the presence of perforation in multiple regressions in elderly patients [36].

*Statement 3*. We recommend against basing the diagnosis of acute appendicitis in elderly patients only on elevated leukocytes count and CRP value. It should prompt adequate diagnostic course (*Strong recommendation, low quality evidence*).

4. What is the optimum pathway for imaging in elderly patients with suspected acute appendicitis? CT or US or both?

When recommending the choice of the imaging strategy, the patients’ age and the potential radiation exposure are important. Although a careful balance of risk-benefit ratio is needed, routine use of CT scan with intravenous (IV) contrast has been demonstrated to be associated with lower negative appendectomy rates [37]. US is inferior to CT in sensitivity and in negative predictive value for appendicitis and may not be as useful for excluding appendicitis [38, 39]. This is particularly true if the appendix is not visualised. False negatives with US are also more likely in patients with a ruptured appendix. Even if, according to Shchatsko et al., the sensitivity of CT scan with IV contrast in diagnosis of acute appendicitis among elderly patients is lower than among general population [5], CT sensitivity, specificity, PPV and NPV for acute appendicitis in patients older than 65 years old reported by other authors are 100%, 99.1%, 95.7% and 100%, respectively [16].

Taking into account that complication rate in elderly patients with negative appendectomy is significantly higher than in younger patients (25% vs 3%, *p* < 0.05) [2], the pre-operative diagnosis in these patients should be as accurate as possible.

For these reasons, the Jerusalem guidelines recommended CT scan with IV contrast in patients older than 60 years old with an Alvarado score ≥ 5 and a negative US [28]. A conditional CT strategy, where CT is performed after a negative US, reduces number of CTs by 50% and correctly identifies as many patients with appendicitis as an immediate CT strategy [28, 40].

Furthermore, in all included studies, elderly patients were significantly more likely than other age groups to have complicated appendicitis with perforation or abscess [2, 4–21].

It is still debated if the prolonged pre-admission delay is associated with an increased perforation rate [7] or not [17]. In fact, according to some studies [17, 41], the duration of symptoms before admission and before operation are not correlated to the risk of perforation. This result is in agreement with the finding, based on epidemiological, immunological and pathological data, according to which acute appendicitis is not a progressive disease, but two types of appendicitis exist: uncomplicated and complicated [42–44]. However, the mortality is significantly higher in elderly patients with perforated appendicitis compared to elderly patients with non-perforated ones (11.9-15% vs 1.52-3%, *p* = 0.0031) [11, 18, 21].

There has not been a clinical trial comparing US and CT scanning to suggest that US can be as accurate as CT in the differentiation of complicated and uncomplicated appendicitis. For ultrasonography, the reported sensitivities for perforated appendicitis vary from 29 to 84% [45, 46].

A meta-analysis by Kim et al. [47] focused on the accuracy of CT scan with IV contrast in distinguishing perforated and non-perforated appendicitis. They found five diagnostic criteria for complicated appendicitis with relatively high pooled diagnostic odds ratios: extraluminal appendicolith, abscess, extraluminal air, appendiceal wall enhancement defect and periappendiceal fat stranding. Each of these criteria individually showed relatively high specificity ranging from 40 to 100%. Periappendiceal fat stranding was the outlier at 40%, the others had a range of 96–100%. These specificities were for individual diagnostic findings, not for additive diagnostic findings.

In a single-center study, Horrow et al. [48] documented that CT criteria for distinction of perforated from non-perforated appendicitis were the presence of a defect in the appendiceal wall, periappendiceal phlegmon or fluid collection, extraluminal air and appendicolith. Apart from periappendiceal phlegmon at a specificity of 94%, these imaging findings all had a specificity of 100% but sensitivities that ranged from 20 (extraluminal appendicolith) to 64% (defect in the enhancing appendiceal wall). However, when a baseline’s set of three criteria (i.e. periappendiceal abscess, extraluminal air, and extraluminal appendicolith) were combined with additional imaging findings of either phlegmon or defect in the appendiceal wall, sensitivities increased to 94% and 96%, respectively.

However, in a study by Hui et al., the introduction of CT scan for the diagnosis of acute appendicitis in elderly patients did not affect outcomes in terms of morbidity and mortality rates [24].

MRI is comparable to US with conditional use of CT with IV contrast in identifying perforated appendicitis. However, both strategies incorrectly classify up to half of all patients with perforated appendicitis as having simple appendicitis [49].

Furthermore, a systematic review to determine the diagnostic test characteristics of non-contrast CT for appendicitis in the adult population found a sensitivity of 92.7% and a specificity of 96.1% [50].

The high prevalence of kidney disease among elderly patients should not discourage the execution of CT scan with IV contrast because most of the time a prompt diagnosis and treatment in this frail population justifies the risk of contrast-induced acute kidney injury (CI-AKI). Furthermore, a recent meta-analysis on retrospective cohort studies of IV radiographic contrast have failed to show a higher risk of CI-AKI after CT scan in patients with chronic kidney disease [51]. The authors proposed that clinicians should reassess the weight attributed to potential CI-AKI in their decision-making process.

In light of these data and balancing risks and benefits, even if the evidences available for elderly patients are undirected and should be classified as low-quality evidences, after the discussion, the panel of experts strongly recommends the use of CT scan in all elderly patients with an Alvarado score ≥ 5 to confirm or exclude the diagnosis of acute appendicitis and to distinguish perforated from non-perforated appendicitis. Due to the frailty of these patients, the panel of experts suggests against discharge elderly patients with an Alvarado score < 5 without an adequate clinical observation. In case of failure to improve a CT scan with IV contrast is suggested.

*Statement 4.1*. We recommend the use of CT scan in all elderly patients with an Alvarado score ≥ 5 to confirm or exclude the diagnosis of acute appendicitis and to distinguish perforated from non-perforated appendicitis (*Strong recommendation, low quality evidence*).

*Statement 4.2*. We suggest that elderly patients with an Alvarado score < 5 should be clinically observed and, in case of failure to improve, they could receive abdominal CT with IV contrast (*Conditional recommendation, very low-quality evidence*).

*Statement 4.3*. We suggest the use of US in elderly patients with an Alvarado score ≥ 5 who cannot undergo CT scan with IV contrast (i.e. acute or chronic kidney disease) to confirm the diagnosis of acute appendicitis, but not for excluding it (*Conditional recommendation, low quality evidence*).

*Statement 4.4*. We suggest against the use of US for distinguishing perforated from non-perforated appendicitis in elderly patients (*Conditional recommendation, very low-quality evidence*).

*Statement 4.5*. We suggest the use of MRI to confirm or exclude the diagnosis of acute appendicitis and to distinguish perforated from non-perforated appendicitis in elderly patients with an Alvarado score ≥ 5 who cannot undergo CT scan with IV contrast (i.e. acute or chronic kidney disease), if this resource is available. If it is not available, non-contrast CT scan is suggested (*Conditional recommendation, very low-quality evidence*).

### Non-operative management

5. Is non-operative management (NOM) feasible for non-complicated acute appendicitis in elderly patients?
  The epidemiologic and clinical studies that elucidate the natural history of appendicitis showed that not all patients with uncomplicated appendicitis will progress to perforation and that spontaneous resolution may be a common event [52]. According to these data, there are two distinct forms of appendicitis: the first one is a mild simple appendicitis that responds to antibiotics or could be even self-limiting, whereas the other often seems to perforate before the patient reaches the hospital [53].
  Several studies showed the feasibility and safety of NOM for uncomplicated appendicitis in general population, with a risk of up to 38% of recurrence [54–56]. According to the Jerusalem guidelines [28] and to a recent review published in the New Engl J Med by Flum [57], appendectomy should be considered the first-line therapy in uncomplicated appendicitis and recommended to the patient, but in the patients with equivocal clinical picture or equivocal imaging, or in those who have strong preferences for avoiding an operation or with major comorbidity or medical problems, it is reasonable to treat with antibiotics first.
  In two recent meta-analysis of RCT comparing appendectomy and NOM on general population, both Pool et al. [58] and Sallinen et al. [59] found that NOM is definitely a feasible and effective treatment for uncomplicated appendicitis, sparing patients from post-operative pain, surgical risk and wound complications. They reported a lower 1 year treatment efficacy [58] and a longer hospital stay [58, 59], but a comparable [58] or lower [59] morbidity and a shorter sick leave duration for NOM compared to appendectomy.
  However, very few data focusing on the safety of NOM in elderly patients exists. In a large Swedish study [25], on more than 117,000 patients, the case fatality rate after appendectomy was strongly influenced by age with a threefold increase for each decade of age, reaching more than 16% in the nonagenarians.
  A retrospective study, based on the National Inpatient Sample (NIS) in the USA [60] focusing on acute appendicitis without peritoneal abscesses, showed an increasing rate of NOM among elderly patients with medical comorbidities who may be perceived as poor operative candidates. However, they found that, after controlling for these factors, patients of all ages who undergo early operative therapy have a decreased risk of mortality. In this study, patients who received percutaneous drainage were excluded.

In a small retrospective study on patients older than 80 years old with non-complicated appendicitis, Park et al. [61] showed that NOM is safe and effective in selected patients, with a NOM success rate higher than 70%.

In light of the absence of high-quality evidences dedicated to the elderly, after discussion, the panel of expert could not make a strong recommendation. NOM is suggested in selected elderly patients with evidence of uncomplicated appendicitis at CT scan, who wish to avoid surgery and accept a risk of recurrence, with a conditional recommendation based on low quality evidences.

*Statement 5*. We suggest the application of NOM in selected elderly patients, with evidence of uncomplicated appendicitis at CT scan and without clinical signs suspected for complicated appendicitis, who wish to avoid surgery and accept the risk of recurrence (*Conditional recommendation, low-quality evidence*).

6. Is NOM with or without percutaneous drainage feasible for complicated acute appendicitis in elderly patients?

The diagnosis of complicated acute appendicitis includes different clinical entities with different clinical behaviours: the well-defined appendicular abscess, the appendicular phlegmon and the free perforated appendicitis with generalised peritonitis. According to Jerusalem guidelines [28] and to recent meta-analysis [62, 63], NOM is a reasonable first-line treatment for appendicitis with phlegmon or abscess and percutaneous drainage, if accessible, is an appropriate treatment in addition to antibiotics. A study on general population [64], focusing on 2209 patients with appendiceal abscesses receiving percutaneous drainage, showed a 74.6% success rate. Older age and later drain placement were predictive of successful treatment with drainage alone. Failure was associated with more charges and longer hospital stay but not with a higher mortality rate. A recent meta-analysis comparing appendectomy and NOM in patients with complicated appendicitis with phlegmon or abscess [65] found lower overall complication, abdominal abscesses, wound infection and unplanned procedures in NOM. A subgroup analysis of three RCT revealed no significant differences in abdominal abscesses and 1-day shorter hospital stay for laparoscopic appendectomy. However, the included studies focused only on young patients (age < 60 years old).

Even in the absence of high-quality evidences, after discussion, in elderly patients with appendicular abscesses percutaneous drainage seems to be the most appropriate treatment. In the case of unavailability of percutaneous drainage or technical impossibility, elderly patients could be treated with antibiotic therapy with strict clinical monitoring. In case of failure to improve or clinical deterioration, laparoscopic abscess drainage and appendectomy should be considered.

In elderly patients with acute appendicitis with free perforation and diffuse peritonitis, as mentioned above, the mortality is significantly higher compared to patients with non-perforated ones (11.9-15% vs 1.5-2.3%, *p* = 0.0031) [11, 18, 21] and these patients require urgent appendectomy.

However, according to most studies [17, 41–44], the delay of the operation is not correlated to the risk of perforation.

*Statement 6.1*. We suggest the use of NOM with percutaneous drainage (if accessible) in elderly patients with complicated appendicitis with appendicular abscess (*Conditional recommendation, low quality evidence*).

*Statement 6.2*. We recommend against the use of NOM in elderly patients with complicated appendicitis with diffuse peritonitis or with a suspected free-perforated appendicitis at CT scan (*Strong recommendation, low quality evidence*).

7. Is colonic screening recommended for elderly patients treated with non-operative management for acute appendicitis?
  The incidence of caecal or appendiceal cancer in patients older than 55-65 years presenting with acute appendicitis ranges from 1.6 to24% [66–68].

The odds ratio of colon cancer incidence had a 38.5-fold increase among patients older than 40 with acute appendicitis [69].

In light of these data and balancing risks and benefits, after the discussion, the panel of experts strongly recommend elective colonic screening in all elderly patients with acute appendicitis, both treated with NOM or appendectomy.

*Statement 7*. We recommend elective colonic screening in all elderly patients with appendicitis (treated both non-operatively and operatively, specially if laparoscopically) (*Strong recommendation, very low-quality evidence*).

### Surgical treatment

8. Should laparoscopic appendectomy be preferred over open appendectomy for elderly patients with acute appendicitis?

When technical skill and equipment are available laparoscopy appendectomy has become the preferred approach to acute appendicitis; guidelines for adult patients recommend the laparoscopic approach in all patients, even in case of complicated acute appendicitis [28]. A recent meta-analysis showed that laparoscopy is associated with longer operative times and higher operative costs, but it leads to less post-operative pain, less surgical site infections, shorter length of stay (LOS) and earlier return to work and physical activity [70].

Several studies investigated the role of laparoscopy in elderly patients, although the definition of elderly patient was not clear. The analysis of the literature available dedicated to elderly patients gave contrasting results with no clear definitions of elderly.

Kirshtein and colleagues retrospectively compared older patients (> 60 years old) who underwent laparoscopic appendectomy with younger patients (< 60): They found similar mortality and morbidity rate with longer LOS in older patients; they also found a significantly higher incidence of complicated acute appendicitis in elderly and a higher rate of complication unrelated to surgical site such as cardiologic complications [71].

Ward et al. retrospectively analysed 257,484 patients older than 65 years who underwent appendectomy in the USA from 1998 to 2009: They found a lower mortality, lower LOS and lower adverse events rate in patients receiving laparoscopic appendectomy [72].

Yeh and colleagues similarly analysed 166,690 patients operated for acute appendicitis: In the subgroups of elderly patients (> 65 years) and patients with comorbidities, laparoscopy was associated with lower length of stay and lower costs rather than open surgery [73].

Southgate summarised the results of all existing studies of open versus laparoscopic appendectomy in elderly patients, and found that laparoscopy is associated with lower mortality, morbidity, costs and length of stay; however, none of the included studies was randomised and it should be noticed that the two study populations were not homogeneous, with higher incidence of complicated appendicitis in open surgery group [74]. At the moment, there are no randomised studies dedicated to elderly patients and more evidences are needed to draw definitive conclusions. At the light of the absence of high-quality evidences dedicated to the elderly, after the discussion, the panel of experts could not make a strong recommendation; laparoscopy is suggested as the preferred technique with a conditional recommendation based on moderate quality evidences.

*Statement 8*. In elderly patients with acute appendicitis, we suggest laparoscopic appendectomy due to a reduced LOS, morbidity and costs (*Conditional recommendation, moderate quality evidences*).

9. In elderly patients operated for acute appendicitis, should the closure of the appendicular stump with linear stapler be preferred over other methods?

The issue of the preferred technique for the closure of appendicular stump is a matter of debate; several studies, even randomised, exist with controversial results. No studies nor subgroup analyses dedicated to elderly patients are available in literature. When compared with endoloop, the use of endostapler seems to be associated with reduced operative time and superficial wound infections rates [75]; otherwise, the two techniques seem not to be different in terms of intra-abdominal abscess, readmission and reoperation rates, with a significantly higher costs associated to the use of endostapler [75–77]. It should be noticed that the evidences available derive from low to moderate quality randomised trials, with no well-designed and underpowered studies included in the meta-analysis. The meta-analysis by Mannu and colleagues analysed and compared also other techniques for the closure of the appendicular stump, such as clips, and even there found no differences. In light of these considerations and of the scarce quality of the existing evidences, moreover with no mention and no specific data about elderly, no strong recommendation could be made for the closure of the appendicular stump; after the discussion, the panel of experts suggests to use the preferred technique based on the local expertise and availability (conditional recommendation based on moderate quality evidences).

*Statement 9*. In elderly patients operated for acute appendicitis, there are no clinical evidences about advantages in the use of linear stapler against other methods (endoloops, clips) for stump closure; we suggest the use of the preferred technique based on local expertise and availability (*Conditional recommendation, moderate quality evidences*.

10. In elderly patients operated for acute appendicitis is the routine placement of a drainage justified?

The use of abdominal drainage after surgical intervention is a controversial matter of debate; it is historically and generally adopted in abdominal sepsis with diffuse peritonitis.

No study dedicated to elderly patients exists; for general population Allemann and colleagues demonstrated, in a case match study on patients with complicated acute appendicitis, that the routine use of drainage was associated with longer LOS and higher complication rate, with similar abdominal abscess rate [78]. Similar findings were confirmed in a meta-analysis of randomised studies: The use of drainage was associated with higher mortality and higher length of stay with similar intra-peritoneal abscess or wound infection rates; all of the included trials were of very low quality and the derived evidences should not allow any solid recommendation [79]. The choice of positioning a drainage after an operation remains an issue of great variability among surgeons, and dedicated and well-designed studies are needed to better analyse the problem. In light of the absence of specific evidences dedicated to elderly patients and of the low quality of the existing evidences about general (adult and paediatric) population, based on the discussion from the panel of experts, in elderly patients, we suggest the use of drainage only in patients with complicated acute appendicitis.

*Statement 10*. In elderly patient, we suggest the positioning of an abdominal drainage in case of complicated (with perforation/abscess/peritonitis) appendicitis (*Conditional recommendation, very low quality evidences*).

11. Does the timing of appendectomy play an important role in elderly patients with acute appendicitis?

The introduction of the conservative treatment as an option for acute appendicitis has raised the question about the timing of surgery and the possible role of delay of surgery. Moreover, not all the hospitals have the availability of an operating room 24/7. From one hand, the initial conservative treatment could decrease the negative explorations rate; from the other, according to some authors, could lead to a delay of surgical treatment of misdiagnosed free perforated appendicitis and consequently to worse outcomes, especially in elderly patients, where diagnosis is more difficult and perforation rate is higher when compared with children and adult population.

A large study by Teixeira and colleagues analysed 4529 patients admitted for suspected acute appendicitis. They found three independent predictors of perforation: age > 55 years, WBC count > 16,000 and female sex, but delay to appendectomy was not associated with higher perforation rate; the delay of operation more than 6 hours was associated with an increase of superficial wound infection rate [80]. Similarly, a large study by Ingraham demonstrated that hospital delay in operation did not affect outcomes: 75% of patients underwent operation within 6 h, 15% between 6 and 12 h and 10% of patients experienced a delay of more than 12 h (mean 26.07 h (SD 132.62)). No clinically significant difference was found in outcomes including overall morbidity and mortality [81]. Differently, Busch et al. reported worse outcomes when appendectomy was postponed more than 12 h: They found as predictors of perforation, the delay of more than 12 h, age over 65 years, time of admission during regular hours, and the presence of comorbidity [82].

Bhangu et al. analysed 2510 patients and found that the delay was not related to complex appendicitis; however, a delay of more than 48 h increased significantly the risk of surgical site infection and adverse events; in the same study, they did a meta-analysis of 11 non-randomised studies including 8858 patients which showed that a delay of 12 to 24 h after admission did not increase the risk of complex appendicitis (OR 0.97, *p* = 0.750) [83].

No dedicated studies to elderly patients exist, but age is indicated in some researches as a risk factor for perforation; since this association, but in absence of clear evidences, after the discussion among the panel of experts, we suggest to perform appendectomy, in elderly patients with operative indication, as soon as possible; however, the level and the quality of the evidence is poor and no strong recommendation could be made.

*Statement 11*. In elderly patient with acute appendicitis, once operation is indicated, we suggest to perform appendectomy as soon as possible (*Conditional recommendation based on very low quality evidences*).

12. Is the removal of the appendix recommended in case of macroscopically normal appendix during abdominal exploration in elderly patients?

Great debate exists about the removal of a normal appendix found during abdominal exploration for a suspected acute appendicitis. Guidelines for adult patients recommend the removal of the appendix with a very low level of evidence and a weak recommendation, based on expert opinions and few controversial evidences (Di Saverio S B. A.).

No data dedicated to this issue in elderly patients are available in literature. Some authors demonstrated that the accuracy of the surgeon in defining a “normal” appendix is very poor with almost apparently normal appendices being inflamed histologically [84]. Similarly, Trong and colleagues confirmed this “inaccuracy” of the surgeon’s judgement with 27.8% of appendix classified as normal and resulted inflamed by histology [85].

On the other hand, the study by Van den Broek et al. demonstrated that leaving a normal looking appendix in absence of other diagnosis in case of abdominal exploration for suspected appendicitis is safe with no complication and a recurrence rate of 6% after a median of 8 months [86]. Moreover, Lee and colleagues demonstrated that the morbidity and complication rate were similar when a normal appendix is removed compared to acute appendicitis [87]. At the light of the contrasting results available in literature and the absence of data dedicated to elderly patients, after an intense debate among participants, no consensus on a statement could be reached and consequently no recommendation could be made.

*Statement 12*. There is no consensus about the removal of a normal appendix with very low quality and indirect evidences; therefore, no recommendation could be made.

### Antibiotic therapy

13. Should the pre-operative antibiotic therapy be recommended before appendectomy in elderly patients?

The rationale of pre-operative antibiotics in acute appendicitis is to reduce and prevent the formation of abdominal abscess and the superficial wound infections rate, similarly to elective surgery. Several studies investigated the issue, but no specific data to elderly patients are available in literature. A meta-analysis including 9576 patients demonstrated that the administration of pre-operative broad-spectrum antibiotics, when compared to no antibiotics, reduced significantly the rate of intra-abdominal abscess and surgical site infection rate [88]. Despite the absence of data dedicated to elderly that forced to downgrade the level of evidences from high to moderate, after the discussion, we decide to make a strong recommendation to use pre-operative antibiotics, due to the large beneficial effect compared to the very low potential harm of the treatment and the extremely unlikelihood that a study dedicated to elderly patients could vary the outcome.

*Statement 13*. We recommend pre-operative broad-spectrum antibiotics in elderly patients undergoing appendectomy for acute appendicitis (*Strong recommendation, moderate quality evidences*).

14. Should post-operative antibiotic therapy be recommended in elderly patients with acute appendicitis?

The issue of the post-operative antibiotic therapy in intra-abdominal infections and appendicitis is largely debated and studied, with several researches published, but no specific data on elderly patients are available.

International guidelines on intra-abdominal infections recommend no post-operative antibiotics in non-complicated intra-abdominal infections [89, 90]; according to these indications, the guidelines about acute appendicitis in general population confirm the recommendation to not continue antibiotics post-operatively when an adequate and effective source control has been obtained [28].

The recommendation was based on several researches and studies; Mui and colleagues randomised patients with non-complicated acute appendicitis to receive only pre-operative, short course or 5 days of antimicrobials: They found that the duration of therapy did not affect the rate of post-operative infections and morbidity [91]. The meta-analysis by Andersen and colleagues demonstrated that, in acute appendicitis, the same outcomes (similar post-operative infection rate) were obtained when no post-operative antibiotics were administrated compared with post-operative therapy [88]. Similarly, in acute cholecystitis, two studies demonstrated that post-operative antibiotic therapy in non-complicated setting, did not reduce the post-operative infection rate [92, 93].

On the contrary, in case of complicated appendicitis, with perforation, abscess or peritonitis, broad spectrum antimicrobial therapy is recommended [89, 90, 94, 95]. Based on these evidences and due to the indirectness of the low quality of them, we suggest administrating antibiotics after the intervention only in case of complicated acute appendicitis or whenever the source control are inadequate (conditional recommendation based on low quality evidence).

*Statement 14.1*. In elderly patients operated on for uncomplicated acute appendicitis, we suggest to not administrate post-operative antibiotics (*Conditional recommendation based on low quality evidences*).

*Statement 14.2*. In elderly patients operated for complicated acute appendicitis, we suggest post-operative broad-spectrum antibiotics (*Conditional recommendation based on low quality evidences*).

15. Should short term post-operative antibiotic therapy be preferred over prolonged therapy after appendectomy in elderly patients?

The duration of antibiotic therapy in intra-abdominal infection is another matter of debate. Few studies dedicated to acute appendicitis exist and no studies dedicated to elderly patients are available. When post-operative antimicrobial therapy is indicated, some studies demonstrated the non-inferiority of limited course of post-operative antibiotics compared to longer therapies: Taylor and colleagues randomised patients with complicated acute appendicitis to receive a minimum of 5 days of post-operative antibiotics versus no indications. In the liberal antibiotic duration group, they demonstrated a less use of antibiotics and the same complication rate [96]. Moreover, the STOP-IT trial demonstrated in complicated intra-abdominal infections, including also appendicitis, that 4 days of antibiotic therapy reached the same outcomes of longer therapies (8 days) with similar morbidity [97]. At the light of these low-quality evidences, due to the lack of data dedicated to elderly, we suggest to continue antibiotic therapy for 3-5 days, although discontinuation of antimicrobial treatment should be based on clinical and laboratory criteria such as fever and leucocytosis.

*Statement 15*. In elderly patients operated for acute appendicitis, when post-operative antibiotic therapy is indicated, we suggest a period of 3-5 days although discontinuation of antimicrobial treatment should be based on clinical and laboratory criteria such as fever and leucocytosis (*Conditional recommendation, low quality evidences*).

## Discussion

AA in elderly patients shows different features and outcomes compared with AA in younger age. After the publication of the Jerusalem guidelines [28] for the diagnosis and management of acute appendicitis in general population, the present guidelines represent, to the best of our knowledge, the first clinical guidelines for diagnosis and management of acute appendicitis in elderly patients.

**Fig. 1:**
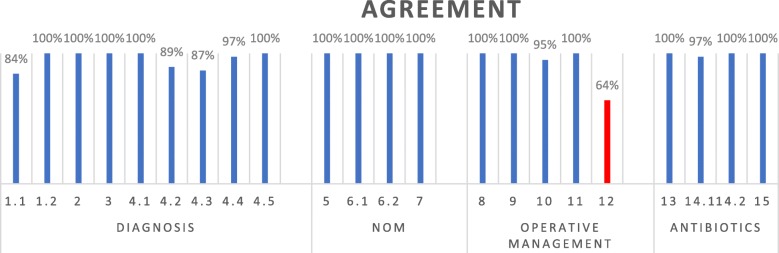
Voting results

**Fig. 2:**
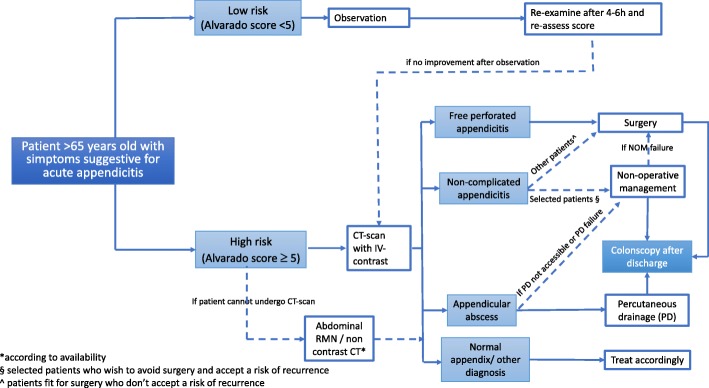
Management algorithm for patients older than 65 years old with suspected AA

Based on the approved statements (Fig. 1 and Table 1), the panel of experts developed a flow-chart diagram for the management of acute appendicitis in the elderly (Fig. 2).

**Table 1:** Statements

Diagnosis	**Statement 1.1** We suggest the use of scoring systems for excluding acute appendicitis in elderly patients with a low-probability score (*Conditional recommendation, low quality evidence*). **Statement 1.2** We suggest against basing the diagnosis of acute appendicitis in elderly patients only on scoring systems (*Conditional recommendation, low quality evidence*).
	**Statement 2** In elderly population, we recommend against basing the diagnosis of acute appendicitis only on patient's clinical signs and symptoms (*Strong recommendation, low quality evidence*).
	**Statement 3** We recommend against basing the diagnosis of acute appendicitis in elderly patients only on elevated leukocytes count and CRP value. It should prompt adequate diagnostic course (*Strong recommendation, low quality evidence*).
	**Statement 4.1** We recommend the use of CT-scan in all elderly patients with an Alvarado score ≥ 5 to confirm or exclude the diagnosis of acute appendicitis and to distinguish perforated from non-perforated appendicitis (*Strong recommendation, low quality evidence*). **Statement 4.2** We suggest that elderly patients with an Alvarado score < 5 should be clinically observed and, in case of failure to improve, they could receive abdominal CT with IV contrast (*Conditional recommendation, very low-quality evidence*). **Statement 4.3** We suggest the use of US in elderly patients with an Alvarado score ≥ 5 who cannot undergo CT scan with IV-contrast (i.e. acute or chronic kidney disease) to confirm the diagnosis of acute appendicitis, but not for excluding it (*Conditional recommendation, low quality evidence*). **Statement 4.4** We suggest against the use of US for distinguishing perforated from non-perforated appendicitis in elderly patients. [*Conditional recommendation, very low-quality evidence].* **Statement 4.5** We suggest the use of MRI to confirm or exclude the diagnosis of acute appendicitis and to distinguish perforated from non-perforated appendicitis in elderly patients with an Alvarado score ≥ 5 who cannot undergo CT scan with IV contrast (i.e. acute or chronic kidney disease), if this resource is available. If it is not available, non-contrast CT scan is suggested (*Conditional recommendation, very low-quality evidence*).
Non-operative management	**Statement 5** We suggest the application of NOM in selected elderly patients, with evidence of uncomplicated appendicitis at CT-scan and without clinical signs suspected for complicated appendicitis, who wish to avoid surgery and accept the risk of recurrence (*Conditional recommendation, low-quality evidence*).
	**Statement 6.1** We suggest the use of NOM with percutaneous drainage (if accessible) in elderly patients with complicated appendicitis with appendicular abscess (*Conditional recommendation, low quality evidence*). **Statement 6.2** We recommend against the use of NOM in elderly patients with complicated appendicitis with diffuse peritonitis or with a suspected free-perforated appendicitis at CT scan (*Strong recommendation, low quality evidence*).
	**Statement 7** We recommend elective colonic screening in all elderly patients with appendicitis (treated both non-operatively and operatively, specially if laparoscopically) (*Strong recommendation, very low-quality evidence*).
Operative management	**Statement 8** In elderly patients with acute appendicitis, we suggest laparoscopic appendectomy due to a reduced LOS, morbidity and costs (*Conditional recommendation, moderate quality evidences*).
	**Statement 9** In elderly patients operated for acute appendicitis, there are no clinical evidences about advantages in the use of linear stapler against other methods (endoloops, clips) for stump closure; we suggest the use of the preferred technique based on local expertise and availability (*Conditional recommendation, moderate quality evidences*).
	**Statement 10** In elderly patient, we suggest the positioning of an abdominal drainage in case of complicated (with perforation/abscess/peritonitis) appendicitis (*Conditional recommendation, very low-quality evidences*).
	**Statement 11** In elderly patient with acute appendicitis, once operation is indicated, we suggest to perform appendectomy as soon as possible (*Conditional recommendation based on very low-quality evidences*).
	**Statement 12** There is no consensus about the removal of a normal appendix with very low quality and indirect evidences; therefore, no recommendation could be made.
Antibiotic therapy	**Statement 13** We recommend pre-operative broad-spectrum antibiotics in elderly patients undergoing appendectomy for acute appendicitis (*Strong recommendation, moderate quality evidences*).
	**Statement 14.1** In elderly patients operated on for uncomplicated acute appendicitis, we suggest to not administrate post-operative antibiotics (*Conditional recommendation based on low quality evidences*). **Statement 14.2** In elderly patients operated for complicated acute appendicitis, we suggest post-operative broad-spectrum antibiotics (*Conditional recommendation based on low quality evidences*).
	**Statement 15** In elderly patients operated for acute appendicitis, when post-operative antibiotic therapy is indicated, we suggest a period of 3-5 days although discontinuation of antimicrobial treatment should be based on clinical and laboratory criteria such as fever and leucocytosis (*Conditional recommendation, low quality evidences*).

The definition of “elderly patients” is one of the most challenging and difficult definition: several criteria could be adopted considering age, clinical conditions, comorbidity, the concept of “biological age” and performance status. Despite the interest in ageing and elderly patients is very high with increasing number of publications about these patients, the concept of “frailty” remains still not clearly defined [98]. Due to a lack of definite criteria and definitions and of well-designed studies in surgical patients with specific including criteria, we decided, according to Pisano et al. [99], to adopt a pragmatic definition of an age older than 65 years to define elderly population, according to the job retirement and life expectancy in Italy and western countries; moreover, most of the available studies in literature adopt this definition. The great limitation of this definition is obvious and clear: age alone could not define the frailty of a patient and patients with the same age could be very different for comorbidity and performance status.

The major part of the statements developed are based on low or very low-quality evidences: this is due to the lack of dedicated studies on elderly (moreover with unclear definition) and to the design of the studies, with the quite impossibility to conduct randomised studies only in elderly patients, and the great difficulty to conduct studies in the field of emergency surgery.

The GRADE methodology forced us to reduce the strength of recommendations, due to the quality of evidence. In fact, strong recommendation could be made only in case of high quality evidences or in very selected cases where, despite the sub-optimal level of evidence, the recommended intervention could be sustained by the likelihood that further research could not change outcomes or the impossibility to demonstrate it with proper studies (i.e. in case of peritonitis conservative treatment cannot be studied due to ethical reasons). The low quality of available evidences highlights the need of further researches dedicated to elderly patients, first of all, with a shared and validated definition of “frail” patients; well-designed studies are needed to overcome these limitations and “fill the gap” in order to reach strong evidence based recommendations.

## Conclusion

As discussed above, the diagnosis of acute appendicitis in elderly remains a clinical challenge due to a very various differential diagnosis; surgical treatment remains the first choice and approach, but not all elderly patients could be “fit for surgery” and different treatment should be evaluated; the non-operative management should be kept in mind with all its well-known limitations and risks (failure and recurrence); moreover, antibiotic treatment in elderly patients, with high probability of MDR pathogens involved in the infection, could become a difficult challenge for the surgeon.

The SIFIPAC along with SIGC, WSES and SIMEU advocate and will promote further studies in order to better study the issue of elderly patient.

## Data Availability

Not applicable

## Abbreviations

AA: Acute appendicitis
AUC: Area under the curve
CRP: C-reactive protein
CT: Computed tomography
ED: Emergency department
IV: Intravenous
LOS: Length of stay
MDR: Multi drug resistant
MRI: Magnetic resonance imaging
NOM: Non-operative management
OR: Odd ratio
SD: Standard deviation
SIFIPAC: Italian Society of Surgical Pathophysiology (SIFIPAC)
SIGC: Italian Society of Geriatric Surgery
SIMEU: Italian Society of Emergency Medicine
US: Ultrasound
WBC: White blood cells
WSES: World Society of Emergency Surgery
